# Explainable Retrospective Sepsis Classification Based on Elixhauser Comorbidity Groups Using Machine and Deep Learning on MIMIC-IV

**DOI:** 10.3390/jcm15155961

**Published:** 2026-07-30

**Authors:** Ying-Chia Wu, Chung-Hsin Lee, Chiung-Chyi Shen, Wen-Yu Cheng, Yi-Chin Yang, Tsung-Wei Wang, Wei-Hsin Hung, Meng-Hsiun Tsai

**Affiliations:** 1 Department of Neurosurgery, Taichung Veterans General Hospital, Taichung 407219, Taiwan; g312985@gmail.com (Y.-C.W.); zacklee228@gmail.com (C.-H.L.); 2 Institute of Medicine, Chung Shan Medical University, Taichung 402367, Taiwan; 3 Department of Neurosurgery, China Medical University Hospital, Taichung 404327, Taiwan; 103755@tool.caaumed.org.tw (C.-C.S.); jean1007@gmail.com (Y.-C.Y.); 4 Department of Post-Baccalaureate Medicine, College of Medicine, National Chung Hsing University, Taichung 402202, Taiwan; wycheng07@yahoo.com.tw; 5 Department of Management Information Systems, National Chung Hsing University, Taichung 402202, Taiwan; twwang19111@gmail.com; 6 Department of Electrical Engineering, National Chung Hsing University, Taichung 402202, Taiwan; d112064007@mail.nchu.edu.tw

**Keywords:** sepsis, comorbidity, retrospective classification, machine learning, explainable artificial intelligence

## Abstract

**Background/Objectives:** Sepsis is clinically heterogeneous, and comorbidities may alter physiological patterns associated with sepsis status. This study examined whether comorbidity-specific modeling revealed differences in retrospective sepsis discrimination. **Methods:** MIMIC-IV data were analyzed across 27 Elixhauser-based analytical subgroups using Random Forest, XGBoost, CatBoost, LightGBM, AdaBoost, multilayer perceptron, TabNet, and FT-Transformer. Available neural-model scripts used a stratified 80/20 training–evaluation split. Class imbalance was addressed using training-partition SMOTE and model-specific loss or sampling settings. SHAP was used to describe model-attributed feature contributions. **Results:** Tree-based ensembles achieved ROC-AUC values above 0.80 in many subgroups, whereas neural models showed more variable performance and trade-offs between discrimination and sepsis-class recall. Red cell distribution width, age, and magnesium were frequently emphasized by the fitted models. **Conclusions:** The findings demonstrate subgroup- and model-specific differences in retrospective sepsis-status discrimination. Because a verified pre-sepsis feature-time boundary was not enforced, the results should not be interpreted as prospectively validated early prediction. External validation, calibration, and decision-analytic evaluation are required before clinical use.

## 1. Introduction

Sepsis is a life-threatening organ dysfunction caused by a dysregulated host response to infection and remains a major global clinical challenge [1]. Survivors may experience substantial long-term health burdens and post-sepsis complications [2,3]. Consistent identification remains challenging because clinical manifestations, disease progression, and treatment responses vary substantially across patients. Early recognition is important for timely intervention; however, the heterogeneity of sepsis limits the reliability of uniform risk-assessment strategies [4,5]. This variability creates a need for models that are both accurate and transparent about the factors contributing to their outputs.

Comorbidity, commonly defined as the coexistence of multiple diseases, should be considered more than a conventional adjustment variable in sepsis research [6]. Chronic conditions may increase baseline vulnerability while also altering immune responses, organ reserve, physiological compensation, and subsequent clinical trajectories [5,7,8]. Consequently, patients with different comorbidity profiles may exhibit distinct biomarker patterns and different relationships between routinely collected clinical variables and sepsis status. Comorbidity-specific analysis may therefore provide information that is obscured when all intensive-care patients are modeled as a single population.

Machine learning and deep learning have been applied to electronic health records for disease risk stratification and outcome prediction [9,10]. Nevertheless, models trained on broad ICU populations may overlook clinically meaningful differences among subgroups, particularly when chronic disease burden modifies both baseline risk and observed laboratory patterns. Moreover, strong discrimination alone does not establish clinical usefulness; transparent outcome definitions, temporally appropriate predictors, calibration, external validation, and assessment of clinical benefit are also required.

The Elixhauser Comorbidity Index (ECI), based on diagnosis codes, provides a structured framework for representing chronic disease burden [11,12]. Although the refined ECI contains 38 categories, the archived analysis reported 27 analytical subgroups. The complete mapping from the refined ECI categories to these 27 subgroups was not retained and therefore could not be independently verified. Existing sepsis studies commonly include comorbidities as covariates, but few studies develop and interpret separate models across comorbidity-defined subgroups [13]. Accordingly, this retrospective study evaluates machine learning and deep learning models within 27 ECI-based analytical subgroups and uses SHAP to characterize subgroup-specific, model-attributed feature patterns. The objective is to examine heterogeneity in retrospective sepsis discrimination rather than to claim a prospectively validated early-warning system.

## 2. Related Research

Comorbidities are closely related to sepsis outcomes and may influence both baseline susceptibility and the ability to tolerate acute physiological stress. Thomas-Rüddel et al. reported that many deaths among ICU patients with sepsis were jointly attributable to sepsis and underlying comorbidities rather than to sepsis alone [14]. This clinical literature supports examining chronic disease profiles as sources of heterogeneous risk patterns rather than treating them only as adjustment variables.

A second body of research has investigated machine learning and deep learning for sepsis-related prediction using electronic health records. Liu et al. developed an interpretable gradient-boosting model using MIMIC-IV emergency data and applied SHAP to describe feature contributions [15]. Systematic and scoping reviews have reported that tree-based ensembles, neural networks, attention mechanisms, and Transformer-based architectures can capture nonlinear relationships in high-dimensional clinical data, while also emphasizing persistent concerns regarding heterogeneity, generalizability, temporal design, and clinical validation [16,17]. These findings do not imply that deep learning universally outperforms conventional approaches; performance depends on cohort size, feature representation, outcome definition, and validation design.

An important remaining gap is the limited evaluation of interpretable models developed separately for comorbidity-defined patient subgroups. The present study addresses this gap by comparing eight machine learning and deep learning algorithms across 27 ECI-based analytical subgroups and by describing model-attributed feature patterns with SHAP. Because the analysis is retrospective and relies on diagnosis-code-based subgroup and outcome information, the results are interpreted as subgroup-specific discrimination and model explanation, not as evidence of causal biological mechanisms or immediate clinical deployment.

## 3. Materials and Methods

This retrospective study used the MIMIC-IV diagnosis, admission, patient, laboratory, and chart-event tables to construct comorbidity-specific analytical datasets. Eight algorithms were compared in the archived results: Random Forest, AdaBoost, XGBoost, CatBoost, LightGBM, multilayer perceptron (MLP), TabNet, and FT-Transformer. The supplied source code allowed one disease-specific cohort-construction script and the MLP, TabNet, and FT-Transformer training configurations to be audited. The available materials did not allow independent verification that the same cohort-construction procedure had been applied consistently to all 27 subgroups. The executable scripts for the five tree-based models and the complete disease-code mapping for all 27 subgroups were not available and are therefore reported as reproducibility limitations.

**Figure 1:**
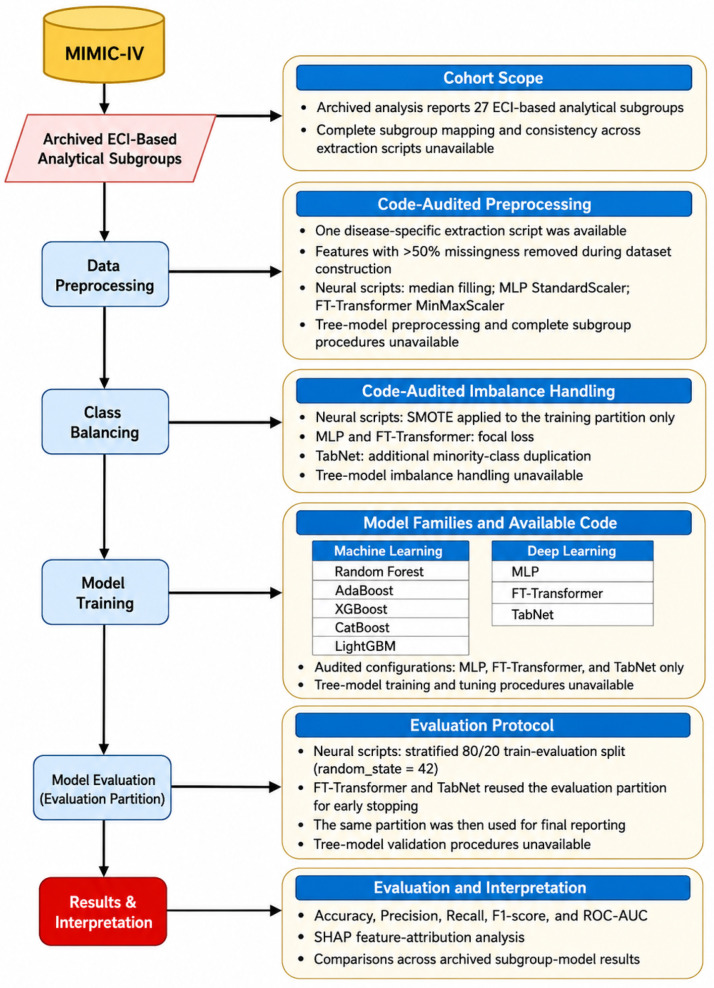
Code-audited retrospective analysis workflow. MIMIC-IV, Medical Information Mart for Intensive Care IV; RF, Random Forest; MLP, multilayer perceptron; SMOTE, Synthetic Minority Over-sampling Technique; SHAP, SHapley Additive exPlanations; ROC-AUC, area under the receiver operating characteristic curve.

Figure 1 summarizes the workflow supported by the available source code.

### 3.1. Cohort Construction and Outcome Definition

The available dataset-construction script merged diagnoses_icd.csv with admission times from admissions.csv. Sepsis-coded records were identified using ICD-9 codes 99591, 99592, and 78552, the ICD-9 prefix 038, and the ICD-10 prefixes A40 and A41. Thus, the outcome was based on administrative diagnosis codes rather than a Sepsis-3 definition using suspected infection and acute organ dysfunction. The first admission time associated with any qualifying sepsis code was treated as the first sepsis-coded admission time.

In the supplied disease-specific extraction script, subjects were assigned to the positive class when the first qualifying comorbidity-coded admission preceded the first sepsis-coded admission. Subjects with the qualifying comorbidity code but no sepsis code in the available diagnosis records were assigned to the negative class. Laboratory and chart-event measurements were retained when their chart time was at or after the first comorbidity-coded admission time, and the earliest value for each item within an admission was selected. The resulting laboratory and chart-event tables were merged by subject, demographic variables were added, and duplicate subject records were removed so that one record per subject was retained. The available materials did not allow independent verification that this procedure had been used for every analytical subgroup.

The extraction code did not impose a verified upper feature-time boundary before the first sepsis-coded admission. Consequently, measurements obtained after the sepsis-coded admission may have entered positive-class feature records. Moreover, admission time linked to an ICD record is not equivalent to the physiological onset time of sepsis. The present task is therefore interpreted as retrospective sepsis-status classification rather than early prediction, and temporal leakage cannot be excluded.

The refined Elixhauser framework contains 38 categories, whereas the archived study reports 27 analytical subgroups. The complete disease-code mapping and all disease-specific extraction scripts were not retained with the available materials. The revised manuscript therefore does not infer an unverified 38-to-27 mapping and acknowledges this omission as a reproducibility limitation. Patients could satisfy more than one comorbidity definition; subgroup analyses are therefore non-mutually exclusive and statistically dependent.

### 3.2. Preprocessing

In the supplied disease-specific extraction script, variables with more than 50% missingness were removed. The three available neural-model scripts then filled remaining numeric missing values using column medians before the training–evaluation split. In addition, the FT-Transformer and TabNet scripts applied a training-fitted median imputer after SMOTE, whereas the MLP script used the initially median-filled table. Because the first median-filling operation was performed before data partitioning, held-out information may have influenced the imputed values; this potential preprocessing leakage is acknowledged as a limitation. Variable-level missingness summaries and sensitivity analyses were not retained.

Preprocessing differed by model. MLP used standardization fitted on the resampled training partition. FT-Transformer used Min–Max scaling fitted on the resampled and imputed training partition, as defined in Equation (1). TabNet used median-imputed numerical inputs without an additional scaling step in the supplied script. The supplied neural-model scripts did not contain the Pearson-correlation feature-selection procedure described in the original manuscript. Therefore, Pearson screening and its equation were removed from the reproducible Methods description rather than being reported as verified steps.

(1) $$x^{\prime }=\frac{x-\min(x)}{\max(x)-\min(x)}$$

### 3.3. Class Balance Handling

**Figure 2:**
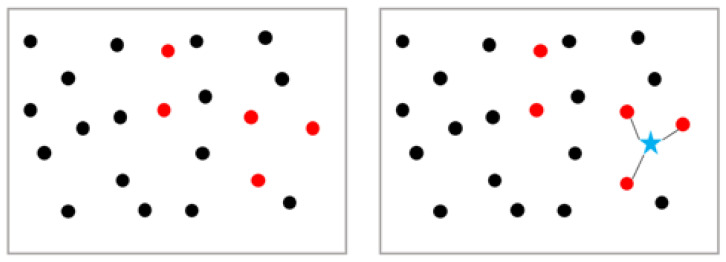
Schematic illustration of Synthetic Minority Over-sampling Technique (SMOTE). Black and red points represent majority- and minority-class observations, respectively, and the blue star represents a synthetic minority observation generated between neighboring minority samples.

As illustrated in Figure 2, all three supplied neural-model scripts applied SMOTE [18] only to the 80% training partition using random_state=42; the held-out 20% partition was not synthetically oversampled. MLP and FT-Transformer used focal loss with γ=2. In the FT-Transformer script, the focal-loss α value was calculated from the resampled training labels. The TabNet script duplicated the minority-class observations once more after SMOTE, producing an additional positive-class emphasis. None of the three supplied neural-model scripts implemented the general class-weight procedure previously stated in the manuscript; that claim was therefore removed. Imbalance-handling details for the five tree-based models could not be independently audited because their scripts were unavailable.

### 3.4. Model Training and Testing

The three available neural-model scripts did not use K-fold cross-validation. Instead, each script divided its input subgroup table using a stratified 80/20 training–evaluation split with test_size=0.2 and random_state=42. The supplied extraction script removed duplicate subject records, making the corresponding row-wise split subject-level; however, consistency of this structure across all subgroup tables could not be independently verified. SMOTE and fitted scaling or imputation operations were applied after the split, except for the initial full-table median filling described above. The three supplied neural-model scripts used fixed configurations rather than an automated hyperparameter-search procedure. The validation and tuning procedures used to generate the five tree-based model results were not available for independent verification.

The five conventional model families represented in the archived results were Random Forest [19], AdaBoost [20], XGBoost [21], CatBoost [22], and LightGBM [23]. The MLP followed the conventional back-propagation paradigm [24] and contained fully connected layers of 64 and 32 units with ReLU activation and a single-logit output. It was trained for 30 epochs with Adam, a learning rate of 0.001, focal loss, a batch size of 64, and a classification threshold of 0.5. The supplied MLP script did not explicitly set a PyTorch random seed.

The FT-Transformer, based on the architecture proposed for tabular data [25], used 64-dimensional tokens, three Transformer blocks, attention dropout of 0.3, feed-forward dropout of 0.2, residual dropout of 0.1, a feed-forward hidden dimension of 128, and a 64-dimensional output representation followed by a binary linear head. It was trained with AdamW at a learning rate of 0.001, a batch size of 128, and a maximum of 100 epochs. Early stopping with patience 10 monitored ROC-AUC on the evaluation partition, and predictions were classified at a threshold of 0.5. The script selected CUDA when available but did not record the hardware used or an explicit PyTorch random seed.

TabNet [26] used $n_{d}=32$, $n_{a}=32$, eight decision steps, γ=1.3, $\lambda_{\mathrm{sparse}}=10^{-4}$, the entmax mask, Adam with a learning rate of 0.01, and a StepLR scheduler with step size 10 and decay factor 0.9. Training used a maximum of 100 epochs, patience 10, batch size 1024, virtual batch size 128, and seed 42. A probability threshold of 0.8 was used for classification. The evaluation partition was supplied as the evaluation set for early stopping.

For FT-Transformer and TabNet, the same evaluation partition was used for early stopping and final performance reporting. This practice can produce optimistic estimates because that partition participates in model selection. Exact package versions and hardware specifications were not preserved. The training scripts used Python with pandas, NumPy, scikit-learn, imbalanced-learn, PyTorch, rtdl, and pytorch-tabnet. Executable configurations for Random Forest, AdaBoost, XGBoost, CatBoost, and LightGBM were not available.

This study evaluated model performance using Precision, Recall, Accuracy, F1-score, and the area under the receiver operating characteristic curve (ROC-AUC). Accuracy was interpreted cautiously because of class imbalance. The definitions of Precision, Recall, Accuracy, and F1-score are as follows:

(2) $$\mathrm{Precision}=\frac{TP}{TP+FP}$$

(3) $$\mathrm{Recall}=\frac{TP}{TP+FN}$$

(4) $$\mathrm{Accuracy}=\frac{TP+TN}{TP+FN+TN+FP}$$

(5) $$F1\text{-}\mathrm{score}=\frac{2\times \mathrm{Precision}\times \mathrm{Recall}}{\mathrm{Precision}+\mathrm{Recall}}$$

F1-macro and ROC-AUC were the primary reported metrics. F1-macro gives equal weight to both classes, whereas ROC-AUC summarizes ranking discrimination across classification thresholds. The definitions of F1-macro, false-positive rate (FPR), and true-positive rate (TPR) are as follows:

(6) $$F1\text{-}\mathrm{macro}=\frac{1}{N}\sum_{i=1}^{N}\frac{2\times {\mathrm{Precision}}_{i}\times {\mathrm{Recall}}_{i}}{{\mathrm{Precision}}_{i}+{\mathrm{Recall}}_{i}}$$

(7) $$\mathrm{FPR}=\frac{FP}{TN+FP}$$

(8) $$\mathrm{TPR}=\frac{TP}{TP+FN}$$

### 3.5. Model Explainability Analysis

Machine learning and deep learning models may provide useful discrimination while remaining difficult to interpret. After model evaluation, SHAP analysis was applied to the selected model within each comorbidity subgroup to describe the magnitude and direction of feature contributions to model outputs. These explanations characterize model behavior within the analyzed dataset; they do not establish that an identified feature causes sepsis or represents a confirmed biological mechanism. SHAP (SHapley Additive exPlanations) [27] was used to quantify model-attributed feature contributions. Based on Shapley values, the method compares predictions across feature subsets and assigns each feature a contribution relative to the model output. The corresponding explainer was selected according to model type. Positive or negative SHAP values indicate the direction in which a feature shifts a prediction relative to the model baseline, whereas aggregated absolute values describe model-level importance. These quantities are predictive explanations and should not be interpreted causally. The SHAP value for feature *i* is defined as follows:

(9) $$\phi_{i}=\sum_{S\subseteq F\setminus \{i\}}\frac{|S|!\left(|F|-|S|-1\right)!}{|F|!}\left[f_{S\cup \{i\}}\left(x_{S\cup \{i\}}\right)-f_{S}\left(x_{S}\right)\right]$$

## 4. Experiments and Results

### 4.1. Classification Performance of Comorbidity Models

This study compared retrospective sepsis discrimination across 27 Elixhauser-based analytical subgroups. F1-macro, ROC-AUC, and sepsis-class Recall were emphasized because subgroup datasets were imbalanced; Accuracy is reported but interpreted cautiously.

Tree-based ensemble methods, particularly CatBoost, LightGBM, and XGBoost, generally provided strong discrimination in many subgroups, although performance varied by comorbidity and metric. Deep learning performance was less uniform. FT-Transformer often produced comparatively high sepsis-class Recall in several subgroups, but it did not consistently outperform ensemble methods in F1-macro or ROC-AUC. For example, in the chronic pulmonary disease subgroup, FT-Transformer achieved an ROC-AUC of 0.7924 and a sepsis Recall of 0.7199, whereas the ensemble models achieved higher ROC-AUC values but lower sepsis Recall. This illustrates the trade-off between ranking performance and minority-class sensitivity rather than universal superiority of a single architecture.

The results are summarized in Table 1, Table 2, Table 3, Table 4 and Table 5. For MLP, TabNet, and FT-Transformer, the supplied scripts used a single stratified 80/20 training–evaluation split rather than K-fold cross-validation. The validation and tuning procedures underlying the Random Forest, AdaBoost, XGBoost, CatBoost, and LightGBM results were not available for independent verification. ROC-AUC denotes the area under the receiver operating characteristic curve, and the Test Samples column reports non-sepsis/sepsis observations in the archived evaluation output. Patient-level predicted probabilities were not retained with the available materials; consequently, ROC-AUC confidence intervals, calibration statistics, and decision-curve analysis could not be reconstructed. For the same reason, Precision–Recall curves and PR-AUC (or average precision) could not be calculated retrospectively from the archived aggregate tables. This omission is particularly important because several analytical subgroups were substantially imbalanced. Future analyses should preserve patient-level scores and report both ROC-AUC and PR-AUC.

### 4.2. Top 10 Comorbidity Models in Retrospective Sepsis Classification

To further examine model performance across different comorbidity categories, models were ranked by F1-macro scores, and the top ten comorbidity-specific models were selected. Recall for positive cases was also incorporated to evaluate overall discriminative ability. Table 6 summarizes the ten subgroup–model combinations with the highest archived F1-macro values. Several of these combinations involved neurological, hematological, psychiatric, or immune-related subgroup labels. This ranking indicates comparatively strong discrimination in the archived evaluation results; it does not establish that the corresponding comorbidities independently increase sepsis risk, nor does it provide an independent statistical comparison among subgroups.

### 4.3. Feature Importance and Model Explainability Analysis

**Figure 3:**
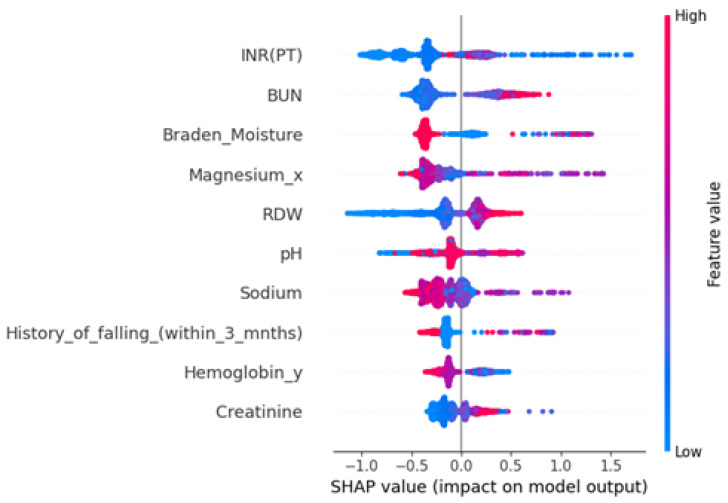
SHAP feature-importance analysis for the Cerebrovascular Disease model. SHAP denotes SHapley Additive exPlanations; larger absolute values indicate greater contribution to the fitted model output and do not imply causality.

**Figure 4:**
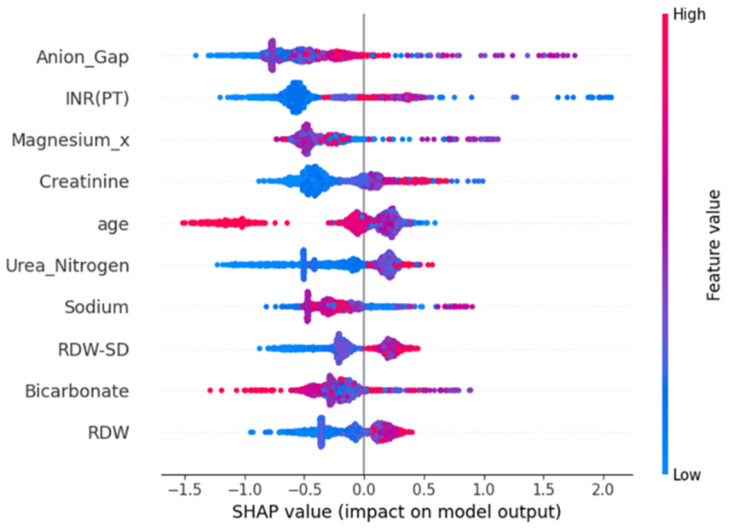
SHAP feature-importance analysis for the Dementia model. SHAP denotes SHapley Additive exPlanations; larger absolute values indicate greater contribution to the fitted model output and do not imply causality.

**Figure 5:**
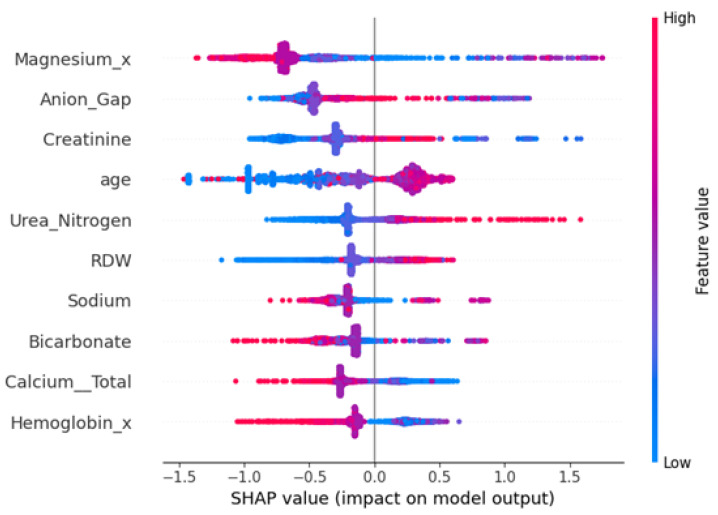
SHAP feature-importance analysis for the Psychoses model. SHAP denotes SHapley Additive exPlanations; larger absolute values indicate greater contribution to the fitted model output and do not imply causality.

**Figure 6:**
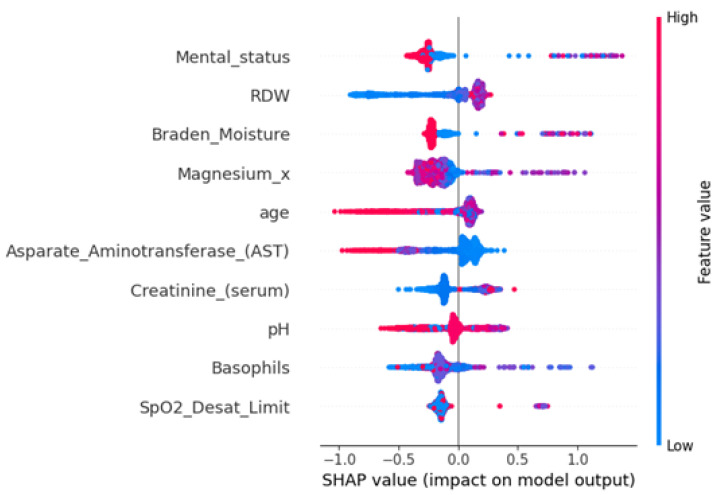
SHAP feature-importance analysis for the Coagulopathy model. SHAP denotes SHapley Additive exPlanations; larger absolute values indicate greater contribution to the fitted model output and do not imply causality.

**Figure 7:**
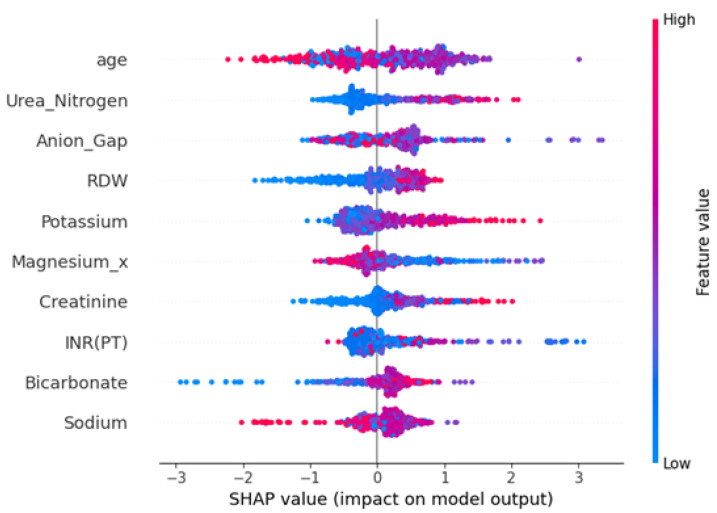
SHAP feature-importance analysis for the Autoimmune Conditions model. SHAP denotes SHapley Additive exPlanations; larger absolute values indicate greater contribution to the fitted model output and do not imply causality.

**Figure 8:**
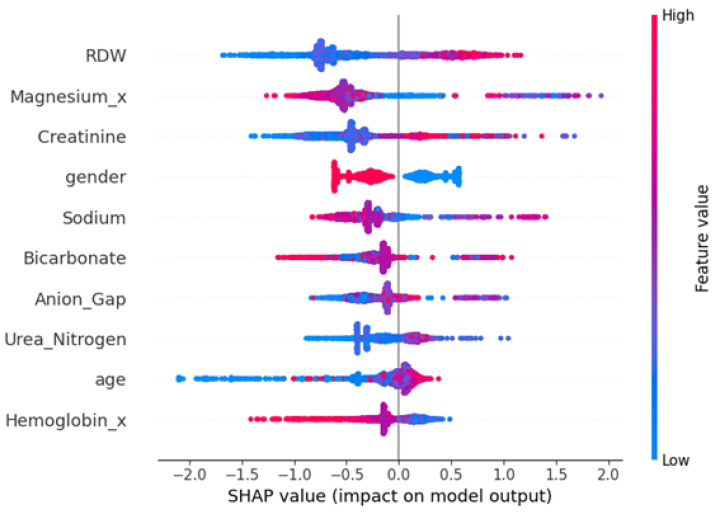
SHAP feature-importance analysis for the Drug Abuse model. SHAP denotes SHapley Additive exPlanations; larger absolute values indicate greater contribution to the fitted model output and do not imply causality.

**Figure 9:**
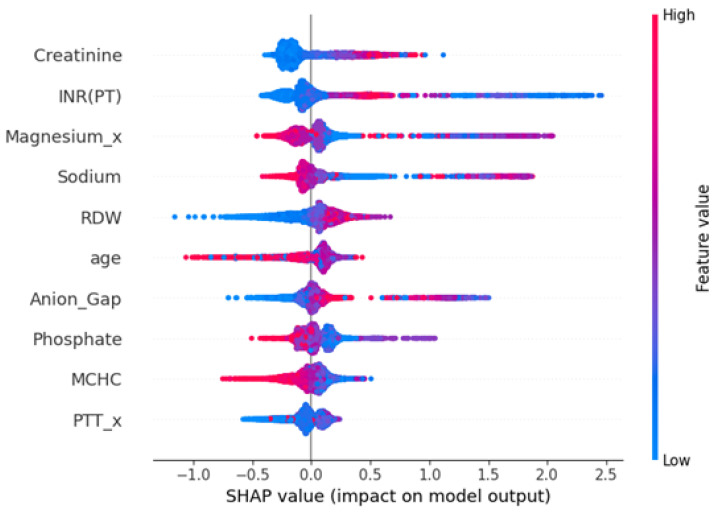
SHAP feature-importance analysis for the Deficiency Anemias model. SHAP denotes SHapley Additive exPlanations; larger absolute values indicate greater contribution to the fitted model output and do not imply causality.

**Figure 10:**
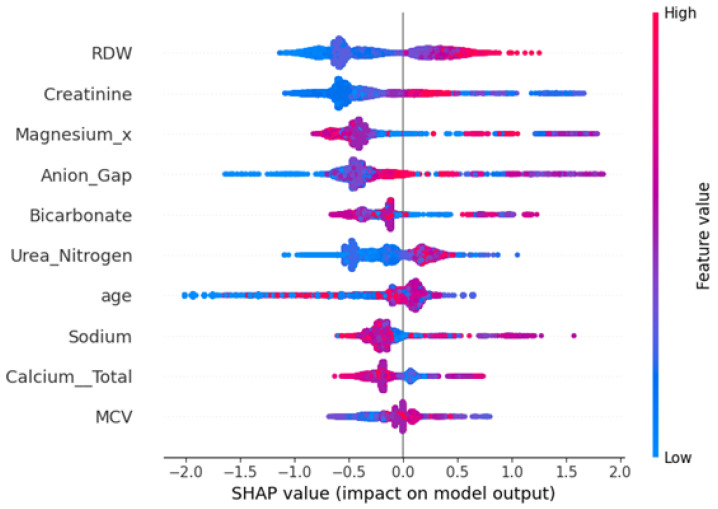
SHAP feature-importance analysis for the Depression model. SHAP denotes SHapley Additive exPlanations; larger absolute values indicate greater contribution to the fitted model output and do not imply causality.

**Figure 11:**
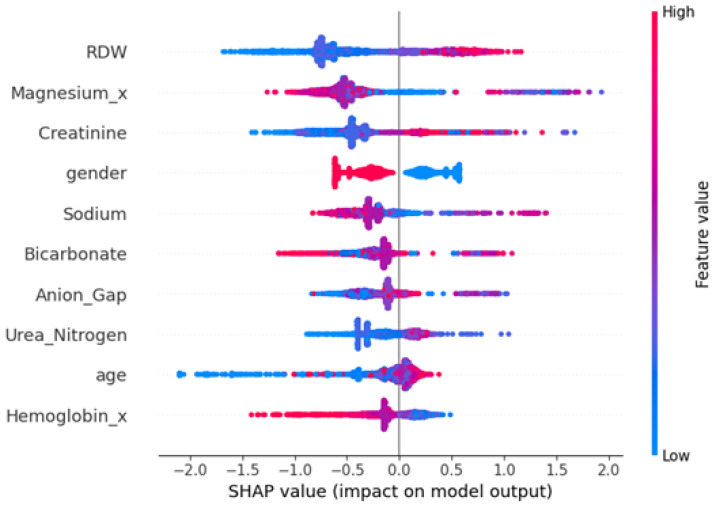
SHAP feature-importance analysis for the Alcohol Abuse model. SHAP denotes SHapley Additive exPlanations; larger absolute values indicate greater contribution to the fitted model output and do not imply causality.

**Figure 12:**
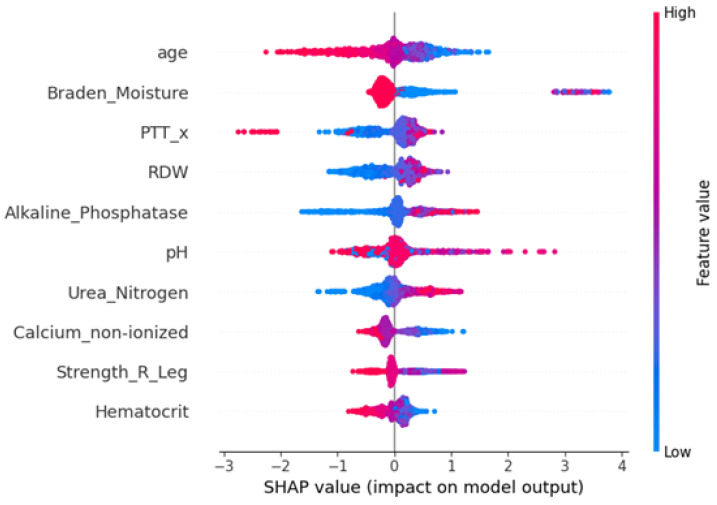
SHAP feature-importance analysis for the Paralysis model. SHAP denotes SHapley Additive exPlanations; larger absolute values indicate greater contribution to the fitted model output and do not imply causality.

To examine the behavior of the comorbidity-specific classifiers, this section presents SHAP feature-importance plots for the ten selected subgroup–model combinations, as shown in Figure 3, Figure 4, Figure 5, Figure 6, Figure 7, Figure 8, Figure 9, Figure 10, Figure 11 and Figure 12. Each plot describes the features emphasized by the fitted model and the direction of their contribution to the model output. These patterns are descriptive model explanations and do not establish physiological mechanisms, clinical effects, or causal relationships.

In each SHAP plot, the horizontal axis represents a feature’s contribution to the model output relative to the model baseline. Values to the right shift the output toward the model’s positive class, whereas values to the left shift it toward the negative class. Point color represents the observed feature value, with red indicating higher values and blue indicating lower values. These directions describe fitted-model behavior and should not be interpreted as changes in biological risk.

## 5. Discussion

The archived results show that classification performance varied across both model architectures and comorbidity-defined analytical subgroups. Tree-based ensemble methods were generally competitive on the structured tabular data, whereas FT-Transformer sometimes produced higher sepsis-class Recall at the cost of lower F1-macro or ROC-AUC. These findings support model- and metric-specific trade-offs rather than universal superiority of deep learning or ensemble learning [28,29]. This pattern is broadly consistent with recent critical-care studies in which gradient-boosting models performed strongly on structured clinical data. Chen et al. reported that LightGBM and XGBoost outperformed several alternatives for ICU sepsis prediction, while also showing a marked reduction in performance after transfer to an external hospital cohort [30]. In a multicenter study of mortality among patients with sepsis, CatBoost, LightGBM, and XGBoost achieved similar discrimination, with only modest differences among the three methods [31]. Aygun et al. likewise found strong performance for XGBoost and LightGBM in an interpretable emergency-department sepsis model [32]. These comparisons support the suitability of boosting methods for tabular ICU data, but they also underscore that apparent within-cohort superiority does not ensure transportability, calibration, or clinical benefit.

The code audit materially changed the interpretation of the validation design. The supplied neural-model scripts used a single stratified 80/20 training–evaluation split with random state 42, not K-fold cross-validation. The validation procedure used for the five tree-based models was not available for independent verification. Moreover, FT-Transformer and TabNet used the evaluation partition for early stopping as well as final reporting. The resulting neural-model performance estimates may therefore be more optimistic and less stable than estimates from nested cross-validation or a separate validation set. The neural-model entries in the reported tables should be interpreted as archived single-split results; the validation design for the tree-based entries remains unverified.

The supplied disease-specific dataset-construction script provides a clearer ICD-based outcome definition: ICD-9 codes 99591, 99592, and 78552, ICD-9 prefix 038, and ICD-10 prefixes A40 and A41. However, this administrative definition is not equivalent to Sepsis-3. More importantly, features were retained after the first comorbidity-coded admission without a verified upper cutoff before the first sepsis-coded admission. Post-sepsis measurements may therefore be present in positive-class records. This design supports retrospective classification of sepsis-coded status but does not support claims of prospective early prediction.

SHAP identified recurring model-attributed features, including red cell distribution width, age, creatinine, magnesium, international normalized ratio/prothrombin time, anion gap, sodium, urea nitrogen, pH, and hemoglobin. Coagulation-related variables are clinically plausible in sepsis because inflammation and coagulation are closely linked [33,34]. Findings in psychiatric and anemia-related subgroups should also be interpreted in the context of the broader clinical complexity of severe mental disorders and chronic iron deficiency [35,36,37]. Previous studies provide clinically plausible associations for several of the remaining variables [38,39,40,41,42]; nevertheless, SHAP values only describe how the fitted model used the variables. They do not demonstrate causality, independent disease effects, or physiological mechanisms.

Patients could satisfy multiple comorbidity definitions, making the subgroup datasets non-mutually exclusive and statistically dependent. Performance differences among subgroups therefore should not be treated as independent head-to-head comparisons. The top-ten ranking describes the strongest archived subgroup–model combinations in the available evaluation results and does not indicate that the corresponding comorbidities independently confer greater sepsis risk.

Several additional limitations remain. First, the complete mapping from the refined Elixhauser categories to all 27 analytical subgroups and the five tree-model training scripts were unavailable. Second, missing values were initially median-filled before the training–evaluation split, which may introduce preprocessing leakage. Third, the MLP and FT-Transformer scripts did not set an explicit PyTorch seed, and exact package versions and hardware specifications were not recorded. Fourth, patient-level predicted probabilities were not retained, preventing valid post hoc ROC-AUC confidence intervals, Precision–Recall analysis, calibration curves, Brier scores, Decision Curve Analysis, net-benefit analysis, or clinical-impact curves. Fifth, threshold selection was not evaluated on an independent validation set; TabNet used a fixed threshold of 0.8. Finally, the study used a single public database and lacks external and prospective validation.

Because clinical implementation depends on both discrimination and the agreement between predicted and observed risks, the current models cannot be considered clinically deployable while their calibration remains unknown. Accordingly, the cautious conclusion reflects not only the temporal and validation concerns described above but also the absence of calibration and decision-analytic evidence. To provide a concise overview, the principal strengths and limitations of the present study are summarized in Table 7 across the domains of study scope, transparency, model evaluation, interpretability, and clinical relevance.

A future reproducible study should rebuild all 27 cohorts using a clinically justified index time; restrict predictors to a prespecified window before Sepsis-3 onset; fit all preprocessing operations using training data only; use patient-level nested cross-validation or separate training, validation, and test sets; preserve patient-level probabilities; report confidence intervals, PR-AUC, and calibration; and evaluate net clinical benefit. All code, disease mappings, package versions, random seeds, and hardware details should be archived.

## 6. Conclusions

This retrospective study compared eight machine learning and deep learning algorithms across 27 Elixhauser-based analytical subgroups in MIMIC-IV and used SHAP to describe model-attributed feature patterns. The available source code confirmed that the neural-model experiments used a single stratified 80/20 training–evaluation split rather than K-fold cross-validation; the validation design for the five tree-based models could not be independently verified. Tree-based ensembles generally showed strong discrimination in the archived results, whereas the neural models displayed variable trade-offs between ROC-AUC and sepsis-class Recall.

The code audit also identified important limitations: ICD-based rather than Sepsis-3 labeling, no verified upper feature-time boundary before the first sepsis-coded admission, preprocessing performed partly before data splitting, reuse of the evaluation partition for early stopping in two models, incomplete reproducibility records, and no retained patient-level probabilities for calibration or decision analysis. Accordingly, the results should be interpreted as retrospective sepsis-status discrimination and hypothesis-generating model explanation, not as a validated early-warning system or a clinically deployable tool.

### Reporting and Reproducibility Note

The revised manuscript reports the procedures that could be verified directly from one supplied disease-specific dataset-construction script and the supplied MLP, TabNet, and FT-Transformer scripts. Details that could not be verified—including the complete 27-subgroup disease mapping, five tree-model scripts, exact software versions, hardware, and patient-level prediction outputs—are explicitly identified as limitations rather than inferred. A fully verified TRIPOD+AI or STROBE-AI checklist would require reconstruction of these missing materials and a new leakage-safe analysis. 

## Figures and Tables

**Table 1:** Classification results of comorbidity models: part I.

Model Name	Accuracy	F1-Macro	ROC-AUC	Non-Sepsis (0) Precision	Non-Sepsis (0) Recall	Sepsis (1) Precision	Sepsis (1) Recall	Test Samples
**Acquired Immune Deficiency Syndrome**								
Random Forest	0.8545	0.7556	0.8226	0.8952	0.9276	0.6522	0.5556	221/29
XGBoost	0.8691	0.7866	0.8368	0.9111	0.9276	0.6800	0.6296	221/29
AdaBoost	0.7927	0.7114	0.7942	0.9100	0.8235	0.4800	0.6667	221/29
CatBoost	0.8727	0.7909	0.8228	0.9115	0.9321	0.6939	0.6296	221/29
LightGBM	0.8764	0.7855	0.8291	0.9013	0.9502	0.7381	0.5741	221/29
MLP	0.6364	0.5797	0.6719	0.8903	0.6244	0.3083	0.6852	221/29
TabNet	0.7236	0.4762	0.5088	0.7984	0.8778	0.1563	0.0926	221/29
FT-Transformer	0.7309	0.6550	0.7700	0.9016	0.7466	0.3913	0.6667	221/29
**Alcohol Abuse**								
Random Forest	0.9096	0.7476	0.8645	0.9438	0.9559	0.5789	0.5156	2723/176
XGBoost	0.9372	0.7884	0.8536	0.9408	0.9923	0.8772	0.4688	2723/176
AdaBoost	0.7621	0.6219	0.8228	0.9600	0.7661	0.2678	0.7281	2723/176
CatBoost	0.9353	0.7834	0.8496	0.9404	0.9905	0.8514	0.4656	2723/176
LightGBM	0.9385	0.7889	0.8564	0.9400	0.9949	0.9130	0.4594	2723/176
MLP	0.7240	0.6259	0.7827	0.9394	0.7235	0.3098	0.7269	2723/176
TabNet	0.8428	0.4957	0.5024	0.8572	0.9791	0.2692	0.0452	2723/176
FT-Transformer	0.7999	0.6986	0.8451	0.9507	0.8076	0.4011	0.7548	2723/176
**Deficiency Anemias**								
Random Forest	0.8908	0.7879	0.8375	0.9014	0.9725	0.8124	0.5282	6695/764
XGBoost	0.9025	0.7999	0.8409	0.8992	0.9916	0.9318	0.5070	6695/764
AdaBoost	0.7850	0.6914	0.8021	0.9091	0.8184	0.4414	0.6368	6695/764
CatBoost	0.9050	0.8023	0.8412	0.8984	0.9963	0.9679	0.5003	6695/764
LightGBM	0.9054	0.8034	0.8411	0.8989	0.9961	0.9669	0.5030	6695/764
MLP	0.6831	0.5999	0.7095	0.8901	0.6978	0.3154	0.6176	6695/764
TabNet	0.8088	0.4618	0.5721	0.8166	0.9875	0.2222	0.0159	6695/764
FT-Transformer	0.6470	0.5754	0.7081	0.8895	0.6479	0.2916	0.6428	6695/764
**Autoimmune Conditions**								
Random Forest	0.8905	0.7930	0.8850	0.9199	0.9508	0.7143	0.5978	447/43
XGBoost	0.9035	0.8051	0.8499	0.9158	0.9732	0.8125	0.5652	447/43
AdaBoost	0.8293	0.7391	0.8458	0.9340	0.8546	0.5000	0.7065	447/43
CatBoost	0.9017	0.8024	0.8487	0.9156	0.9709	0.8000	0.5652	447/43
LightGBM	0.9072	0.8085	0.8446	0.9144	0.9799	0.8500	0.5543	447/43
MLP	0.8089	0.7107	0.7965	0.9236	0.8389	0.4586	0.6630	447/43
TabNet	0.8275	0.4632	0.5866	0.8302	0.9955	0.3333	0.0109	447/43
FT-Transformer	0.6939	0.6166	0.7714	0.9222	0.6890	0.3220	0.7174	447/43
**Chronic Blood Loss (Iron Deficiency)**								
Random Forest	0.9368	0.8559	0.9340	0.9389	0.9902	0.9186	0.6309	2559/174
XGBoost	0.9415	0.8666	0.9263	0.9415	0.9930	0.9414	0.6465	2559/174
AdaBoost	0.9019	0.8088	0.9012	0.9450	0.9394	0.6645	0.6868	2559/174
CatBoost	0.9424	0.8687	0.9252	0.9419	0.9937	0.9477	0.6488	2559/174
LightGBM	0.9441	0.8717	0.9305	0.9420	0.9957	0.9635	0.6488	2559/174
MLP	0.8990	0.5832	0.6394	0.9471	0.9449	0.2167	0.2241	2559/174
TabNet	0.7768	0.5940	0.6680	0.8831	0.8503	0.2934	0.3557	2559/174
FT-Transformer	0.8525	0.5853	0.7305	0.9552	0.8839	0.1863	0.3908	2559/174
**Cancer**								
Random Forest	0.8959	0.7402	0.8089	0.9032	0.9828	0.8061	0.4049	4816/586
XGBoost	0.8943	0.7130	0.7502	0.8936	0.9940	0.9068	0.3310	4816/586
AdaBoost	0.7719	0.6178	0.7058	0.8958	0.8279	0.3188	0.4554	4816/586
CatBoost	0.8952	0.7130	0.7527	0.8933	0.9956	0.9300	0.3275	4816/586
LightGBM	0.8964	0.7196	0.7643	0.8950	0.9948	0.9206	0.3404	4816/586
MLP	0.6815	0.5922	0.6885	0.8749	0.7087	0.3112	0.5651	4816/586
TabNet	0.8070	0.4758	0.5229	0.8141	0.9875	0.3750	0.0321	4816/586
FT-Transformer	0.5660	0.5214	0.6695	0.8814	0.5372	0.2577	0.6898	4816/586

**Table 2:** Classification results of comorbidity models: part II.

Model Name	Accuracy	F1-Macro	ROC-AUC	Non-Sepsis (0) Precision	Non-Sepsis (0) Recall	Sepsis (1) Precision	Sepsis (1) Recall	Test Samples
**Cerebrovascular Disease**								
Random Forest	0.9336	0.8549	0.9307	0.9377	0.9871	0.9003	0.6399	2941/220
XGBoost	0.9362	0.8604	0.9238	0.9390	0.9888	0.9132	0.6474	2941/220
AdaBoost	0.8933	0.7968	0.8852	0.9383	0.9354	0.6514	0.6623	2941/220
CatBoost	0.9416	0.8720	0.9297	0.9416	0.9925	0.9416	0.6623	2941/220
LightGBM	0.9390	0.8660	0.9278	0.9397	0.9915	0.9332	0.6511	2941/220
MLP	0.8991	0.6283	0.6935	0.9493	0.9419	0.2963	0.3273	2941/220
TabNet	0.8596	0.7418	0.8482	0.9251	0.9075	0.5405	0.5970	2941/220
FT-Transformer	0.8807	0.5518	0.7238	0.9378	0.9337	0.1631	0.1727	2941/220
**Coagulopathy**								
Random Forest	0.9060	0.8060	0.8892	0.9046	0.9899	0.9188	0.5231	1580/171
XGBoost	0.9076	0.8146	0.8870	0.9099	0.9848	0.8889	0.5549	1580/171
AdaBoost	0.8437	0.7426	0.8300	0.9118	0.8962	0.5603	0.6040	1580/171
CatBoost	0.9071	0.8138	0.8731	0.9099	0.9842	0.8848	0.5549	1580/171
LightGBM	0.9060	0.8106	0.8888	0.9083	0.9848	0.8873	0.5462	1580/171
MLP	0.8458	0.5715	0.6449	0.9167	0.9120	0.2235	0.2339	1580/171
TabNet	0.7150	0.5213	0.5249	0.8281	0.8234	0.2141	0.2197	1580/171
FT-Transformer	0.5604	0.5566	0.6306	0.9133	0.9203	0.2075	0.1930	1580/171
**Dementia**								
Random Forest	0.9193	0.8279	0.8695	0.9319	0.9757	0.8229	0.6128	1276/98
XGBoost	0.9305	0.8458	0.8598	0.9321	0.9898	0.9167	0.6085	1276/98
AdaBoost	0.8491	0.7452	0.8488	0.9367	0.8809	0.5113	0.6766	1276/98
CatBoost	0.9318	0.8480	0.8532	0.9322	0.9914	0.9286	0.6085	1276/98
LightGBM	0.9318	0.8460	0.8633	0.9303	0.9937	0.9459	0.5957	1276/98
MLP	0.7028	0.6091	0.7486	0.9241	0.7061	0.3004	0.6851	1276/98
TabNet	0.8438	0.4817	0.4652	0.8471	0.9945	0.4615	0.0255	1276/98
FT-Transformer	0.7723	0.6666	0.8034	0.9291	0.7908	0.3718	0.6723	1276/98
**Depression**								
Random Forest	0.9252	0.7730	0.8777	0.9429	0.9754	0.7075	0.5017	7487/489
XGBoost	0.9390	0.7954	0.8744	0.9414	0.9936	0.8983	0.4780	7487/489
AdaBoost	0.8011	0.6495	0.8384	0.9553	0.8157	0.3034	0.6776	7487/489
CatBoost	0.9396	0.7925	0.8776	0.9398	0.9963	0.9359	0.4611	7487/489
LightGBM	0.9398	0.7939	0.8765	0.9401	0.9961	0.9342	0.4645	7487/489
MLP	0.7028	0.6091	0.7486	0.9241	0.7061	0.3004	0.6851	7487/489
TabNet	0.8410	0.4773	0.5835	0.8540	0.9814	0.1775	0.0233	7487/489
FT-Transformer	0.7154	0.6294	0.8414	0.9526	0.7013	0.3143	0.7970	7487/489
**Diabetes**								
Random Forest	0.8777	0.7176	0.7960	0.9052	0.9568	0.6285	0.4219	4579/530
XGBoost	0.8961	0.7262	0.7946	0.8999	0.9880	0.8410	0.3665	4579/530
AdaBoost	0.7862	0.6509	0.7695	0.9145	0.8264	0.3563	0.5542	4579/530
CatBoost	0.8961	0.7258	0.7951	0.8998	0.9882	0.8430	0.3652	4579/530
LightGBM	0.8982	0.7306	0.7993	0.9006	0.9897	0.8622	0.3703	4579/530
MLP	0.7190	0.6287	0.7339	0.8918	0.7449	0.3525	0.6057	4579/530
TabNet	0.8136	0.4496	0.5847	1.0000	0.8972	1.0000	0.0010	4579/530
FT-Transformer	0.6777	0.6119	0.7537	0.9104	0.6698	0.3310	0.7124	4579/530
**Drug Abuse**								
Random Forest	0.9286	0.7705	0.8781	0.9482	0.9741	0.6784	0.5066	2124/117
XGBoost	0.9435	0.8045	0.8496	0.9498	0.9896	0.8429	0.5153	2124/117
AdaBoost	0.7845	0.6330	0.8397	0.9644	0.7905	0.2729	0.7293	2124/117
CatBoost	0.9426	0.8005	0.8527	0.9490	0.9896	0.8406	0.5066	2124/117
LightGBM	0.9418	0.7955	0.8415	0.9477	0.9901	0.8433	0.4934	2124/117
MLP	0.7578	0.6473	0.8012	0.9436	0.7646	0.3280	0.7155	2124/117
TabNet	0.8114	0.5716	0.5967	0.8796	0.9049	0.2786	0.2287	2124/117
FT-Transformer	0.7882	0.6828	0.8330	0.9546	0.7919	0.3713	0.7654	2124/117

**Table 3:** Classification results of comorbidity models: part III.

Model Name	Accuracy	F1-Macro	ROC-AUC	Non-Sepsis (0) Precision	Non-Sepsis (0) Recall	Sepsis (1) Precision	Sepsis (1) Recall	Test Samples
**Heart Failure**								
Random Forest	0.9023	0.7382	0.8701	0.9068	0.9876	0.8385	0.3884	4993/552
XGBoost	0.9043	0.7561	0.8695	0.9132	0.9818	0.7996	0.4379	4993/552
AdaBoost	0.8622	0.7125	0.7974	0.9165	0.9235	0.5171	0.4934	4993/552
CatBoost	0.9062	0.7577	0.8671	0.9128	0.9848	0.8253	0.4331	4993/552
LightGBM	0.9074	0.7623	0.8729	0.9141	0.9846	0.8266	0.4427	4993/552
MLP	0.8301	0.5868	0.6372	0.9214	0.8870	0.2358	0.3152	4993/552
TabNet	0.8036	0.6816	0.7728	0.8888	0.8688	0.4622	0.5090	4993/552
FT-Transformer	0.8857	0.5597	0.6963	0.9094	0.9696	0.3153	0.1268	4993/552
**Hypertension**								
Random Forest	0.8834	0.7135	0.7856	0.9026	0.9679	0.6741	0.3884	5459/628
XGBoost	0.8986	0.7237	0.7839	0.8998	0.9918	0.8797	0.3530	5459/628
AdaBoost	0.7836	0.6436	0.7443	0.9127	0.8256	0.3448	0.5376	5459/628
CatBoost	0.9003	0.7210	0.7878	0.8981	0.9963	0.9403	0.3380	5459/628
LightGBM	0.9000	0.7251	0.7921	0.8997	0.9938	0.9058	0.3509	5459/628
MLP	0.7441	0.6539	0.7449	0.9024	0.7694	0.3831	0.6327	5459/628
TabNet	0.7773	0.5465	0.6026	0.8300	0.9141	0.3133	0.1731	5459/628
FT-Transformer	0.6681	0.6054	0.7623	0.9145	0.6541	0.3233	0.7298	5459/628
**Liver Disease**								
Random Forest	0.8575	0.7081	0.7941	0.8871	0.9489	0.6264	0.4150	1995/298
XGBoost	0.8691	0.6980	0.7585	0.8777	0.9784	0.7650	0.3398	1995/298
AdaBoost	0.7212	0.6103	0.7084	0.8903	0.7569	0.3179	0.5485	1995/298
CatBoost	0.8720	0.6926	0.7488	0.8747	0.9870	0.8333	0.3155	1995/298
LightGBM	0.8741	0.7035	0.7556	0.8780	0.9850	0.8225	0.3374	1995/298
MLP	0.6538	0.5945	0.6919	0.8760	0.6551	0.3320	0.6490	1995/298
TabNet	0.7879	0.4425	0.5696	0.7906	0.9955	0.1000	0.0019	1995/298
FT-Transformer	0.6677	0.6043	0.7078	0.8764	0.6752	0.3421	0.6395	1995/298
**Chronic Pulmonary Disease**								
Random Forest	0.9213	0.7659	0.8329	0.9356	0.9787	0.7379	0.4713	6844/505
XGBoost	0.9335	0.7841	0.8376	0.9342	0.9952	0.9224	0.4495	6844/505
AdaBoost	0.7964	0.6427	0.8023	0.9444	0.8187	0.3040	0.6216	6844/505
CatBoost	0.9335	0.7823	0.8387	0.9335	0.9961	0.9346	0.4427	6844/505
LightGBM	0.9323	0.7792	0.8351	0.9331	0.9950	0.9187	0.4404	6844/505
MLP	0.7427	0.6354	0.7594	0.9235	0.7589	0.3290	0.6529	6844/505
TabNet	0.7860	0.5995	0.6720	0.8786	0.8670	0.3153	0.3382	6844/505
FT-Transformer	0.7117	0.6202	0.7924	0.9334	0.7103	0.3103	0.7199	6844/505
**Neurological Disorders**								
Random Forest	0.9206	0.7709	0.8484	0.9412	0.9715	0.6888	0.5094	2141/39
XGBoost	0.9314	0.7756	0.8253	0.9352	0.9916	0.8676	0.4453	2141/39
AdaBoost	0.8246	0.6645	0.8171	0.9460	0.8515	0.3361	0.6075	2141/39
CatBoost	0.9327	0.7781	0.8307	0.9353	0.9930	0.8872	0.4453	2141/39
LightGBM	0.9339	0.7827	0.8219	0.9362	0.9935	0.8955	0.4528	2141/39
MLP	0.6930	0.6032	0.7783	0.9329	0.6880	0.2909	0.7211	2141/39
TabNet	0.8346	0.5475	0.6199	0.8610	0.9603	0.3609	0.1263	2141/39
FT-Transformer	0.7069	0.6164	0.7909	0.9370	0.7020	0.3043	0.7342	2141/39
**Obesity**								
Random Forest	0.9108	0.7535	0.8575	0.9327	0.9688	0.6703	0.4760	4834/385
XGBoost	0.9259	0.7695	0.8374	0.9292	0.9915	0.8723	0.4341	4834/385
AdaBoost	0.8230	0.6677	0.8093	0.9401	0.8537	0.3508	0.5922	4834/385
CatBoost	0.9266	0.7701	0.8429	0.9290	0.9928	0.8882	0.4310	4834/385
LightGBM	0.9264	0.7706	0.8414	0.9293	0.9921	0.8805	0.4341	4834/385
MLP	0.7357	0.6415	0.7752	0.9309	0.7412	0.3384	0.7064	4834/385
TabNet	0.7662	0.6323	0.7214	0.8995	0.8132	0.3409	0.5155	4834/385
FT-Transformer	0.7875	0.6722	0.8023	0.9193	0.8196	0.3902	0.6159	4834/385

**Table 4:** Classification results of comorbidity models: part IV.

Model Name	Accuracy	F1-Macro	ROC-AUC	Non-Sepsis (0) Precision	Non-Sepsis (0) Recall	Sepsis (1) Precision	Sepsis (1) Recall	Test Samples
**Paralysis**								
Random Forest	0.9332	0.7813	0.8919	0.9355	0.9933	0.8939	0.4504	1051/75
XGBoost	0.9349	0.7887	0.8720	0.9372	0.9933	0.8971	0.4656	1051/75
AdaBoost	0.9019	0.7494	0.8461	0.9440	0.9458	0.5581	0.5496	1051/75
CatBoost	0.9340	0.7870	0.8814	0.9371	0.9924	0.8841	0.4656	1051/75
LightGBM	0.9306	0.7761	0.8844	0.9353	0.9905	0.8551	0.4504	1051/75
MLP	0.9023	0.5636	0.6520	0.9410	0.9553	0.2034	0.1600	1051/75
TabNet	0.8336	0.5248	0.5737	0.8565	0.9657	0.3208	0.0909	1051/75
FT-Transformer	0.9139	0.6231	0.7745	0.9483	0.9600	0.3226	0.2667	1051/75
**Peripheral Vascular Disease**								
Random Forest	0.8920	0.7339	0.8091	0.9135	0.9659	0.6740	0.4350	2613/279
XGBoost	0.9022	0.7359	0.8114	0.9089	0.9851	0.8088	0.3901	2613/279
AdaBoost	0.7668	0.6342	0.7746	0.9231	0.7953	0.3185	0.5910	2613/279
CatBoost	0.9071	0.7445	0.8139	0.9097	0.9904	0.8691	0.3924	2613/279
LightGBM	0.9051	0.7413	0.8099	0.9095	0.9881	0.8426	0.3924	2613/279
MLP	0.7155	0.6236	0.7400	0.8991	0.7363	0.3381	0.6197	2613/279
TabNet	0.8183	0.4666	0.5378	0.8229	0.9923	0.3333	0.0176	2613/279
FT-Transformer	0.7108	0.6296	0.7601	0.9115	0.7176	0.3434	0.6796	2613/279
**Psychoses**								
Random Forest	0.9289	0.7830	0.8913	0.9556	0.9663	0.6411	0.5726	2228/117
XGBoost	0.9480	0.8148	0.8570	0.9522	0.9924	0.8786	0.5256	2228/117
AdaBoost	0.8087	0.6571	0.8779	0.9695	0.8142	0.2995	0.7564	2228/117
CatBoost	0.9509	0.8236	0.8761	0.9531	0.9946	0.9124	0.5342	2228/117
LightGBM	0.9488	0.8150	0.8294	0.9515	0.9942	0.9030	0.5171	2228/117
MLP	0.8340	0.7211	0.8478	0.9518	0.8510	0.4344	0.7265	2228/117
TabNet	0.8558	0.4865	0.4664	0.8656	0.9861	0.2439	0.0285	2228/117
FT-Transformer	0.7736	0.6670	0.8341	0.9541	0.7751	0.3485	0.7635	2228/117
**Pulmonary Circulation Disease**								
Random Forest	0.9006	0.7359	0.7881	0.9194	0.9709	0.6864	0.4280	1822/172
XGBoost	0.9107	0.7433	0.7756	0.9169	0.9868	0.8182	0.3985	1822/172
AdaBoost	0.8146	0.6577	0.7620	0.9248	0.8568	0.3556	0.5314	1822/172
CatBoost	0.9102	0.7390	0.7889	0.9156	0.9879	0.8268	0.3875	1822/172
LightGBM	0.9126	0.7407	0.7747	0.9149	0.9918	0.8729	0.3801	1822/172
MLP	0.7173	0.6300	0.7364	0.9185	0.7239	0.3355	0.6846	1822/172
TabNet	0.8258	0.4698	0.5680	0.8321	0.9901	0.2800	0.0189	1822/172
FT-Transformer	0.7433	0.6454	0.7696	0.9133	0.7634	0.3567	0.6442	1822/172
**Renal Failure and Disease**								
Random Forest	0.7965	0.6490	0.7216	0.8419	0.9141	0.5221	0.3533	1258/258
XGBoost	0.8273	0.6656	0.7166	0.8411	0.9634	0.6954	0.3144	1258/258
AdaBoost	0.7255	0.6293	0.7123	0.8585	0.7814	0.3848	0.5150	1258/258
CatBoost	0.8266	0.6543	0.7175	0.8368	0.9698	0.7164	0.2874	1258/258
LightGBM	0.8279	0.6604	0.7035	0.8388	0.9682	0.7143	0.2994	1258/258
MLP	0.6151	0.5740	0.6650	0.8319	0.6137	0.3432	0.6195	1258/258
TabNet	0.7542	0.4323	0.6023	0.7545	0.9992	0.5000	0.0024	1258/258
FT-Transformer	0.5857	0.5558	0.6615	0.8363	0.5604	0.3297	0.6634	1258/258
**Thyroid Disorders**								
Random Forest	0.9205	0.7658	0.8555	0.9363	0.9768	0.7240	0.4776	5266/388
XGBoost	0.9314	0.7788	0.8417	0.9338	0.9932	0.8925	0.4463	5266/388
AdaBoost	0.8209	0.6637	0.8189	0.9444	0.8481	0.3372	0.6075	5266/388
CatBoost	0.9299	0.7733	0.8475	0.9327	0.9926	0.8825	0.4373	5266/388
LightGBM	0.9318	0.7775	0.8452	0.9330	0.9945	0.9102	0.4388	5266/388
MLP	0.7561	0.6494	0.7759	0.9277	0.7721	0.3464	0.6674	5266/388
TabNet	0.7622	0.4969	0.5482	0.8462	0.8788	0.1493	0.1175	5266/388
FT-Transformer	0.8009	0.6980	0.8239	0.9394	0.8177	0.4128	0.7083	5266/388

**Table 5:** Classification results of comorbidity models: part V.

Model Name	Accuracy	F1-Macro	ROC-AUC	Non-Sepsis (0) Precision	Non-Sepsis (0) Recall	Sepsis (1) Precision	Sepsis (1) Recall	Test Samples
**Peptic Ulcer with Bleeding**								
Random Forest	0.8596	0.6655	0.7597	0.8902	0.9525	0.5476	0.3286	800/96
XGBoost	0.8840	0.6964	0.7712	0.8931	0.9813	0.7541	0.3286	800/96
AdaBoost	0.7574	0.6284	0.7512	0.9121	0.7913	0.3211	0.5643	800/96
CatBoost	0.8840	0.6964	0.7643	0.8931	0.9813	0.7541	0.3286	800/96
LightGBM	0.8894	0.7011	0.7594	0.8928	0.9888	0.8333	0.3214	800/96
MLP	0.6944	0.6138	0.6910	0.8929	0.7088	0.3343	0.6324	800/96
TabNet	0.7482	0.5326	0.5235	0.8232	0.8788	0.2595	0.1838	800/96
FT-Transformer	0.7208	0.6348	0.7552	0.8959	0.7425	0.3602	0.6270	800/96
**Valvular Disease**								
Random Forest	0.9209	0.7731	0.8947	0.9270	0.9873	0.8367	0.4556	2521/223
XGBoost	0.9240	0.7838	0.8863	0.9295	0.9881	0.8507	0.4750	2521/223
AdaBoost	0.8875	0.7366	0.8449	0.9323	0.9397	0.5529	0.5222	2521/223
CatBoost	0.9195	0.7734	0.8856	0.9282	0.9841	0.8077	0.4667	2521/223
LightGBM	0.9236	0.7796	0.8902	0.9279	0.9897	0.8646	0.4611	2521/223
MLP	0.8317	0.4817	0.7016	0.8381	0.9897	0.3659	0.0302	2521/223
TabNet	0.7710	0.5382	0.5804	0.8466	0.8866	0.2434	0.1851	2521/223
FT-Transformer	0.9034	0.5788	0.7685	0.9286	0.9695	0.3125	0.1570	2521/223
**Weight Loss**								
Random Forest	0.8628	0.6825	0.7482	0.8831	0.9640	0.6443	0.3378	1919/261
XGBoost	0.8799	0.7022	0.7524	0.8845	0.9854	0.8146	0.3324	1919/261
AdaBoost	0.7837	0.6388	0.7191	0.8912	0.8452	0.3667	0.4649	1919/261
CatBoost	0.8772	0.6905	0.7556	0.8813	0.9865	0.8156	0.3108	1919/261
LightGBM	0.8838	0.7003	0.7567	0.8825	0.9937	0.9063	0.3135	1919/261
MLP	0.7523	0.6348	0.6963	0.8603	0.8244	0.3971	0.4635	1919/261
TabNet	0.7723	0.4784	0.4902	0.8012	0.9515	0.2185	0.0543	1919/261
FT-Transformer	0.7548	0.6536	0.7507	0.8749	0.8093	0.4125	0.5365	1919/261

**Table 6:** Metrics of top 10 comorbidity models.

Comorbidity Group	Accuracy	F1-Macro	ROC-AUC	Non-Sepsis (0) Precision	Non-Sepsis (0) Recall	Sepsis (1) Precision	Sepsis (1) Recall
Cerebrovascular Disease	0.9416	0.8720	0.9297	0.9416	0.9925	0.9416	0.6623
Dementia	0.9318	0.8480	0.8532	0.9322	0.9914	0.9286	0.6085
Psychoses	0.9509	0.8236	0.8761	0.9531	0.9946	0.9124	0.5342
Coagulopathy	0.9076	0.8146	0.8870	0.9099	0.9848	0.8889	0.5549
Autoimmune Conditions	0.9072	0.8085	0.8446	0.9144	0.9799	0.8500	0.5543
Drug Abuse	0.9435	0.8045	0.8496	0.9498	0.9896	0.8429	0.5153
Deficiency Anemias	0.9054	0.8034	0.8411	0.8989	0.9961	0.9669	0.5030
Depression	0.9390	0.7954	0.8744	0.9414	0.9936	0.8983	0.4780
Alcohol Abuse	0.9385	0.7889	0.8564	0.9400	0.9949	0.9130	0.4594
Paralysis	0.9349	0.7887	0.8720	0.9372	0.9933	0.8971	0.4656

**Table 7:** Summary of the principal strengths and limitations of the study.

Domain	Strengths	Limitations
Study scope	Comparison of eight machine-learning and deep-learning methods across 27 archived Elixhauser-based analytical subgroups.	Subgroups were overlapping, the complete 38-to-27 mapping was unavailable, and the analysis used a single public database.
Transparency	A code-based audit distinguished verified procedures from unsupported statements and reframed the task as retrospective classification.	Five tree-model scripts, exact software versions, hardware details, and complete intermediate cohort outputs were not retained.
Model evaluation	Multiple discrimination and class-specific metrics were reported, together with archived subgroup class counts.	The neural models used a single split; some evaluation data were reused for early stopping; confidence intervals, PR-AUC, calibration, and net benefit could not be reconstructed.
Interpretability	SHAP provided subgroup-specific descriptions of model-attributed feature contributions.	SHAP findings are predictive associations and do not establish causality, independent disease effects, or biological mechanisms.
Clinical relevance	The analysis highlights heterogeneity in model behavior across clinically recognizable comorbidity groups.	No verified pre-sepsis feature window, Sepsis-3 labeling, external validation, prospective testing, or calibration assessment was available.

## Data Availability

The data analyzed in this study are available from the MIMIC-IV database on PhysioNet at https://physionet.org/content/mimiciv/. Access is restricted to credentialed users who complete the required training and sign the applicable Data Use Agreement. The data cannot be redistributed by the authors.
